# Disruptions of Autophagy in the Rat Retina with Age During the Development of Age-Related-Macular-Degeneration-like Retinopathy

**DOI:** 10.3390/ijms20194804

**Published:** 2019-09-27

**Authors:** Oyuna S. Kozhevnikova, Darya V. Telegina, Mikhail A. Tyumentsev, Nataliya G. Kolosova

**Affiliations:** Institute of Cytology and Genetics, Siberian Branch of Russian Academy of Sciences (SB RAS), Pr. Lavrentyeva 10, Novosibirsk 630090, Russia

**Keywords:** autophagy, age-related macular degeneration, retina, retinal pigment epithelium, transcriptome, chloroquine, fasting

## Abstract

Age-related macular degeneration (AMD) is one of the main causes of vision impairment in the elderly. Autophagy is the process of delivery of cytoplasmic components into lysosomes for cleavage; its age-related malfunction may contribute to AMD. Here we showed that the development of AMD-like retinopathy in OXYS rats is accompanied by retinal transcriptome changes affecting genes involved in autophagy. These genes are associated with kinase activity, immune processes, and FoxO, mTOR, PI3K-AKT, MAPK, AMPK, and neurotrophin pathways at preclinical and manifestation stages, as well as vesicle transport and processes in lysosomes at the progression stage. We demonstrated a reduced response to autophagy modulation (inhibition or induction) in the OXYS retina at age 16 months: expression of genes *Atg5*, *Atg7*, *Becn1*, *Nbr1*, *Map1lc3b*, *p62*, and *Gabarapl1* differed between OXYS and Wistar (control) rats. The impaired reactivity of autophagy was confirmed by a decreased number of autophagosomes under the conditions of blocked autophagosome–lysosomal fusion according to immunohistochemical analysis and transmission electron microscopy. Thus, the development of AMD signs occurs against the background of changes in the expression of autophagy-related genes and a decrease in autophagy reactivity: the ability to enhance autophagic flux in response to stress.

## 1. Introduction

Age-related macular degeneration (AMD) is a multifactorial retinal neurodegenerative disease, one of major causes of severe irreversible vision loss in the elderly worldwide [1]. Aging is the main AMD risk factor. A complex interaction of multiple genetic and environmental factors leads to degeneration of photoreceptors, of the retinal pigment epithelium (RPE), and of Bruch’s membrane, as well as alterations in choroidal capillaries. Many studies have established a link between the dysregulation of complement, lipid, angiogenic, inflammatory, oxidative-stress, and extracellular-matrix pathways and AMD pathogenesis [1]. Furthermore, there is increasing evidence that defective proteostasis due to impaired clearance might be the key process in AMD [2]. Nonetheless, researchers are still far from precise knowledge of the etiology of this disease. Molecular studies on AMD are hampered by the inaccessibility of live retinal tissue from AMD patients, especially in the early stages [3]. According to clinical manifestations and pathological changes, AMD is subdivided into two forms: dry AMD (atrophic) and wet AMD (exudative). A total of 80% of AMD patients have a diagnosis of the dry form of AMD, for which no effective treatment exists today [2]. During dry AMD, the loss of vision primarily involves progressive degeneration of macular RPE cells under the influence of the accumulation of lipofuscin and drusen; these alterations subsequently exert adverse effects on neurosensory layers of the retina [4]. Recent studies proved the critical role of macroautophagy in the homeostasis of aging RPE cells [5,6,7]. 

Macroautophagy (hereafter autophagy) is an evolutionarily conserved catabolic process crucial for cellular homeostasis because autophagy involves the sequestration, delivery, and degradation of damaged or unneeded proteins, macromolecular complexes, and organelles into lysosomes [8]. The autophagy–lysosomal system is implicated in protein quality control, and its dysregulation impairs proteostasis, one of the primary hallmarks of aging [9]. Autophagy is rapidly upregulated in response to a variety of stressors, including starvation, organelle or DNA damage, hypoxia, endoplasmic-reticulum stress, or infection [10]. Increasing numbers of studies have shown that autophagy participates in the pathogenesis of many age-related neurodegenerative diseases. Uncovering the involvement of autophagy in these diseases is essential because it may point to a novel therapeutic target [11]. Autophagy stimulation may result in reduced accumulation of misfolded and aggregated proteins; however, the overactivation of autophagy can trigger autophagy-mediated apoptosis [10]. Conversely, suppression of autophagy could lead to the accumulation of cellular and ultimately extracellular waste like lipofuscin, amyloid, and atherosclerotic plaques. 

Of particular importance for the functioning of the RPE is a noncanonical form of autophagy termed LC3-associated phagocytosis, in which components of the autophagy pathway converge with phagocytosis to recover vitamin A in the visual cycle [12]. RPE cells constantly phagocytose used photoreceptors’ outer segments (POSs), this process is followed by the autophagy–lysosomal degradation of POSs [4]. Proper removal of POSs by RPE cells is extremely important to maintain the function of photoreceptors [13]. It can be assumed that disturbances in LC3-associated phagocytosis in the RPE can cause accumulation of intracellular aggregates, including lipofuscin, and promote the formation of drusen, typical for AMD, thereby triggering subsequent degeneration of underlying neurosensory cells.

Research on the molecular mechanisms underlying the age-related dysregulation of autophagy at the early stage of AMD and before its development can give some clues to the most relevant molecular events triggering the entry into the irreversible stage. We have shown that senescence-accelerated OXYS rats spontaneously reproduce the major signs of AMD and stages of the disease: dystrophic alterations of the RPE, thinning of the neuroretina, and impairment of choroidal microcirculation [14,15,16,17]. Retinopathy that emerges in OXYS rats already at 3 month of age corresponds mostly to the dry atrophic form of AMD. Nonetheless, with age, the presence of fluid or retinal hemorrhage, known hallmarks of wet AMD, is seen in some (~10–20%) of rats. The first clinical signs of the AMD-like retinopathy in OXYS rats (a combination of multiple small and a few medium-size drusen-like deposits) develop by the age of 3 months simultaneously with a reduction in the transverse area of the RPE and impairment of choroidal microcirculation [18,19,20]. With age, retinal degenerative changes in OXYS rats increase, accompanied by excessive accumulation of lipofuscin in RPE regions, disturbances in the morphology of the RPE sheet (including an increase in the proportion of multinucleated cells), hypertrophy, distortion of the cell shape, and reactive gliosis by 12 months and pronounced outer-retina thinning by 18 months of age [15,21]. Previously, we have demonstrated that the autophagic pathway participates in the progression of AMD-like retinopathy in OXYS rats [16]. One of the consequences of autophagy impairment may be significant accumulation of a toxic drusen component (amyloid β fragment, Aβ_1–42_) in the aged retina of OXYS rats [17,18].

Here, we analyzed high-throughput RNA sequencing (RNA-Seq) data to identify the specific molecular processes and pathways that take part in the alterations of autophagy during the development and progression of AMD-like retinopathy in OXYS rats. On the basis of previous results revealing an important role of autophagy, we explored the in vivo effects of autophagy activation and inhibition by fasting and chloroquine (CQ) treatment, respectively, on the expression of autophagy markers in the retina of OXYS and control Wistar rats.

## 2. Results

### 2.1. Differentially Expressed Genes (DEGs) Associated with Autophagy in OXYS Rats

**Figure 1:**
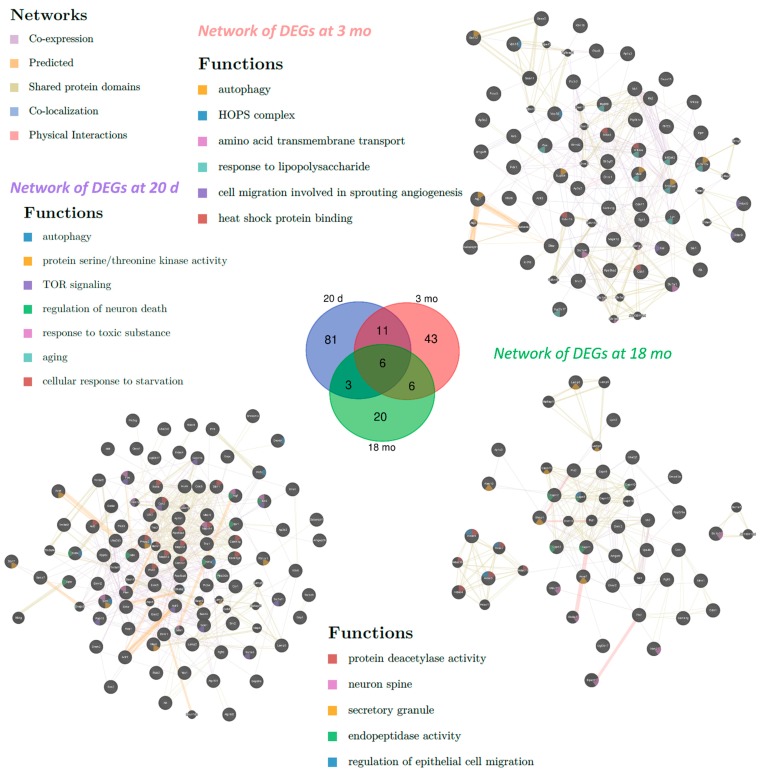
Interaction networks of differentially expressed genes (DEGs) involved in autophagy in the retina of OXYS rats compared with Wistar rats at 20 days and 3 and 18 months of age. In the center, the Venn diagram illustrates overlapping sets of DEGs in OXYS rats relative to Wistar rats at various ages.

To identify the pathways and biological functions involved in the alteration of autophagy in OXYS rats, we analyzed RNA-Seq data obtained previously [22,23]. Using databases DAVID (Database for Annotation, Visualization and Integrated Discovery) and AUTOPHAGY, we compiled lists of the differentially expressed genes (DEGs) associated with autophagy in the retina of 20-day-old and 5- and 18-month-old OXYS rats compared to age-matched Wistar rats. We found 101 DEGs at 20 days, 66 DEGs at 3 months, and 35 DEGs at 18 months of age in OXYS rats relative to age-matched Wistar rats (Figure 1, Table S1). In OXYS rats, changes in the expression of six autophagy-associated genes (*Hdac9*, *Lamp3*, *Camk1g*, *Ehmt2*, *Tbc1d5*, and *Ugt2b17*) were detectable at all the analyzed ages. Eleven autophagy-related DEGs were common for the ages 20 days and 3 months, six for the ages 3 and 18 months, and three for the ages 20 days and 18 months. Functional annotation of the DEGs (according to DAVID) in the retina of OXYS rats compared to Wistar rats at 20 days of age yielded gene ontology (GO) terms associated with protein phosphorylation, intracellular signal transduction, negative regulation of TOR signaling, TORC2 signaling, histone deacetylation, regulation of cell proliferation, and neuronal cell death (Table 1). As for molecular functions, these genes were associated with the following terms: serine/threonine kinase and MAP kinase activity, ATP binding, protein deacetylation, and transcription factor and phosphatase binding. In terms of cellular components, enrichment was revealed for the Golgi apparatus, endosome membrane, TORC2 complex, and neuron projection. At the age of 20 days, DEGs were found to be associated with the following signaling pathways: FoxO, mTOR, PI3K-AKT, neurotrophin, sphingolipid, MAPK, AMPK, Toll-like receptor, and TNF. Clustering analysis showed that clusters with scores higher than 3 were three groups of genes: possessing kinase activity (enrichment score (ES): 6.69), regulation of autophagy (ES: 3.62), and included in the signaling pathways of FoxO, MAPK, and neurotrophins (ES: 3.46).

Functional analysis of autophagy-related DEGs at an early stage of retinopathy (3 months) indicated that their protein products were associated with protein phosphorylation, innate immune response, negative regulation of apoptosis, intracellular signal transduction and protein transport, response to oxidative stress, regulation of proliferation, and activity of NF-κB. Regarding molecular functions, these DEGs were associated with kinase activity, transcription factor binding, and ATP. In terms of cellular components, enrichment for the lysosome, early endosome, late endosome, and AP-3 complex was revealed. At the age of 3 months, autophagy-related DEGs turned out to be associated with several signaling pathways: neurotrophin, Toll-like receptor, B cell receptor, FoxO, T cell receptor, TNF, and lysosome (Table 1). Clustering analysis suggested that clusters with scores above 3 were groups of genes with kinase activity (ES: 3.95) and related to the immune system (ES: 3.6). 

At the late stage of retinopathy (18 months), protein products of autophagy-related DEGs in the retina of OXYS rats were associated with the development and differentiation of neurons, neurogenesis, the regulation of the organization of the cellular component, organelle division, stress response, vesicle transport, and synaptic signaling. In the category of molecular functions, these DEGs were found to be associated with calcium-dependent cysteine-endopeptidase activity, hydrolase, kinase and phosphotransferase activity, as well as binding of purine nucleotides. In terms of cellular components, enrichment was identified for the lysosome, endosome, Golgi apparatus, focal adhesion, and postsynaptic density. Among Kyoto Encyclopedia of Genes and Genomes (KEGG) pathways at this age, enrichment for the endocytosis pathway was detected (Table 1). Clusterization analysis showed that the cluster with the highest score (ES: 3.46) was a group of genes (*Capn1*, *Capn2*, *Capn3*, and *Capn5*) encoding thiol proteases. Their expression was lower in OXYS rats.

We employed GeneMANIA to construct interaction networks for the analysis of DEGs involved in autophagy in the retina of OXYS rats compared with Wistar rats at 20 days and 3 and 18 months of age (Figure 1). It was evident that most of the genes were directly or indirectly related to one another. For DEGs between OXYS and Wistar rats at 20 days, GO terms were among the most enriched and included autophagy, protein serine/threonine kinase activity, TOR signaling, cellular response to starvation, aging, response to toxic substance, and regulation of neuron death. Among the GO terms enriched in the set of DEGs at 3 months of age, there were autophagy, HOPS complex, amino acid transmembrane transport, response to lipopolysaccharide, and cell migration involved in angiogenesis, as well as heat shock protein binding. For the set of DEGs at 18 months, we uncovered the following enriched GO terms: regulation of epithelial cell migration, protein deacetylase activity, endopeptidase activity, secretory granule, and neuron spine.

As for age-related changes in gene expression, from 3 to 18months, 33 DEGs were detected in Wistar rats and 45 in OXYS rats; among them, only seven were common for both rat strains (Table S1). A total of 38 DEGs were typical for OXYS rats: 20 genes were upregulated, and 18 genes were downregulated. Analysis of autophagy-related DEGs that changed expression from 3 to 18 months in OXYS rats uncovered their connection with the processes of intracellular signal transduction, phosphorylation, vesicle transport, regulation of proliferation and activity of NF-κB, neuron differentiation, and signaling pathways of MAPK and neurotrophins. Genes that changed expression with age in Wistar rats turned out to be associated with protein phosphorylation, apoptosis, MAPKK cascade, and organelle division.

Of note, at all ages, the expression of genes that regulate the fusion of membrane structures in the cell, including the fusion of autophagosomes with lysosomes, was found to change in the retina of OXYS rats. First of all, these include Rab proteins from the family of small GTPases that control vesicular traffic as well as components of the SNARE complex. Recently, it was reported that inhibition of the SNARE complex disrupts autophagic flux, judging by the accumulation of mature autophagosomes that cannot merge with lysosomes [24]. Rab proteins facilitate the formation of autophagic vacuoles and regulate the clearance of aggregated proteins by autophagy [25,26]. From the “fusion regulation” group, the expression of 18 genes was lower at 20 days of age in OXYS rats (e.g., *Tbc1d15*, *Usp6nl*, *Gdi2*, *Rab10*, *Rab11a*, *Rab14*, *Rab21*, *Rab2a*, *Rab30*, and *Rab6a*), and the expression of eight genes was higher (*Rabl6*, *Rab36*, *Tbc1d10b*, *Tbc1d32*, *Tbc1d5*, *Vps52*, *Grtp1*, and *Ift22*) in comparison with the Wistar strain. At 3 months of age in OXYS rats, the expression of five genes was lower (*Rab15*, *Rab27b*, *Rab30*, *Rab7b*, and *Usp6nl*), and 10 genes were found to be upregulated (*Rabl6*, *Rab36*, *Tbc1d32*, *Tbc1d13*, *Grtp1*, *Hps4*, *Nbas*, *Vps39*, *Vps18*, and *Stx3*). At 18 months in OXYS rats, the expression of six genes was lower (*Rab10*, *Rab11a*, *Rab15*, *Rab27b*, *Usp6nl*, and *Vps52*), and that of five genes was higher (*Vps50*, *Rab36*, *Tbc1d32*, *Tbc1d5*, and *Grtp1*) in comparison with the Wistar strain. From 3 to 18 months of age in OXYS rats, the expression of *Rabl3*, *Abi1*, *Scfd1*, *Tmem41b*, *Vps39*, and *Vps32* decreased, and that of *Exoc3l4*, *Syn2*, *Syt9*, *Rabif*, and *Tbc1d30* increased. It can be assumed that such changes in the expression of genes that regulate the fusion of vesicles and control vesicle trafficking could reflect the impaired autophagic flux in the retina of OXYS rats.

### 2.2. The Effects of Autophagy Modulation on the Expression of Autophagy Markers 

Next, we studied changes in the mRNA levels of genes characteristic of different stages of autophagy in the retina in an experiment on autophagy modulation (blocking or induction). Autophagy inhibition was implemented by the administration of chloroquine (CQ), which blocks the stage of fusion of autophagosomes with lysosomes [8].

The effects of autophagy modulation were studied by monitoring of changes in the expression of autophagy-related genes depending on various factors: genotype (Wistar versus OXYS) or exposure to starvation (for 12, 24, or 48 h) and CQ (in combination with starvation). For this analysis, we chose the genes whose products participate at different stages of autophagy: *Becn1* (initiation), *Atg5* and *Atg7* (phagophore formation), *Map1lc3b* (hereinafter *Lc3b*) and *Gabarapl1* (elongation and maturation of autophagosomes), and *p62* also known as *Sqstm1* (hereinafter *p62*) and *Nbr1* (selective autophagy). At the age of 4 months, analysis of variance (ANOVA) revealed changes in the expression of genes *Atg7*, *Gabarapl1*, and *Lc3b*. Exposure to CQ increased Atg7 expression in Wistar rats but not in the OXYS strain (*p* < 0.05). The mRNA expression of *Gabarapl1* depended on the genotype and was higher in OXYS rats (*p* < 0.05). Lc3b levels also depended on the genotype and were higher in Wistar rats (*p* < 0.05). For genes *Atg5*, *Becn1*, *Nbr1*, and *p62*, no significant differences in expression between the two groups were detected at age 4 months.

**Figure 2:**
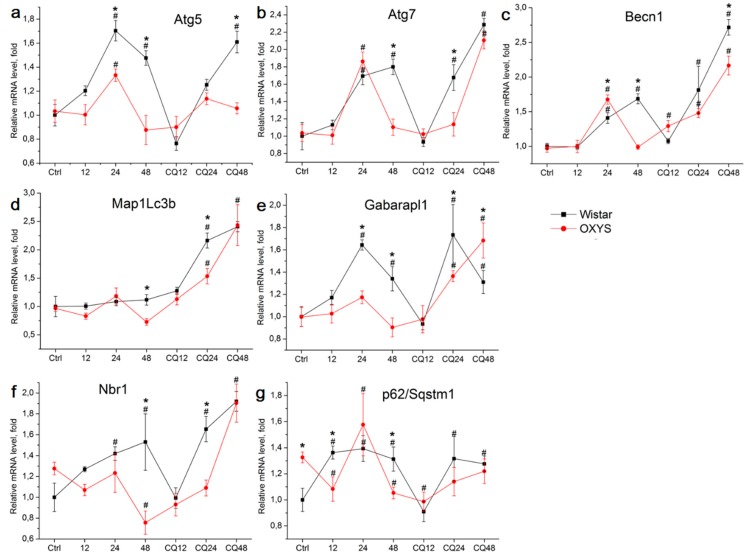
The effects of fasting or fasting plus chloroquine (CQ) treatment on mRNA levels of genes *Atg5* (**a**), *Atg7* (**b**), *Becn1* (**c**), *Map1Lc3b* (**d**), *Gabarapl1* (**e**), *Nbr1* (**f**), and *p62* (**g**) in the retina of 16-month-old OXYS and Wistar (control strain) rats. Differences in the dynamics of expression of autophagy markers during fasting indicate lowered reactivity of the autophagy system in OXYS rats. Data are presented as mean ± SD, *n* = 6. *Significant differences between the strains; ^#^significant differences from control animals of the same strain.

Significant differences between the groups were noted at the age of 16 months for all the chosen genes (Figure 2). Dispersion analysis indicated that the mRNA levels of all genes (except for p62) depended on the genotype and treatment, and the factors “genotype” and “treatment” interacted. Thus, in response to autophagy modulation, the expression of the studied genes at the transcriptional level differed between OXYS and Wistar rats. By 12 h of fasting, the *p62* mRNA level increased in Wistar rats and decreased in OXYS rats. By 24 h of fasting, *Atg5*, *Atg7*, and *Becn1* mRNA expression increased in the rats of both strains, where the *Atg5* mRNA level was higher in Wistar rats, while the *Becn1* mRNA level was higher in OXYS rats. Furthermore, by 24 h of fasting, the expression of *Gabarapl1* and *Nbr1* increased in Wistar rats, as was the case for *p62* in OXYS rats. In contrast to Wistar rats, by 48 h of fasting in OXYS rats, *Atg5*, *Atg7*, and *Becn1* mRNA levels decreased to control values. In Wistar rats, *Atg5* mRNA expression slightly decreased by 48 h but remained above the control value, *Atg7* expression remained as high as that at 24 h, and *Becn1* mRNA expression became higher than that at 24 h. By 48 h of fasting in Wistar rats, *Gabarapl1* mRNA expression decreased but remained above the control level. By 48 h, mRNA levels of *Nbr1* and *p62*, markers of selective autophagy, decreased below the control values in OXYS rats, while remaining high in Wistar rats. Significant interstrain differences were registered in changes in the mRNA levels of genes *Atg5*, *Atg7*, *Becn1*, *Gabarapl1*, *Map1lc3b*, and *Nbr1* in response to inhibition of autophagy by CQ. The CQ treatment neutralized the effects of 24 h fasting by shifting the peak of mRNA upregulation from 24 to 48 h (Figure 2).

### 2.3. Decreased Autophagic Reactivity in the Retina of OXYS Rats 

**Figure 3:**
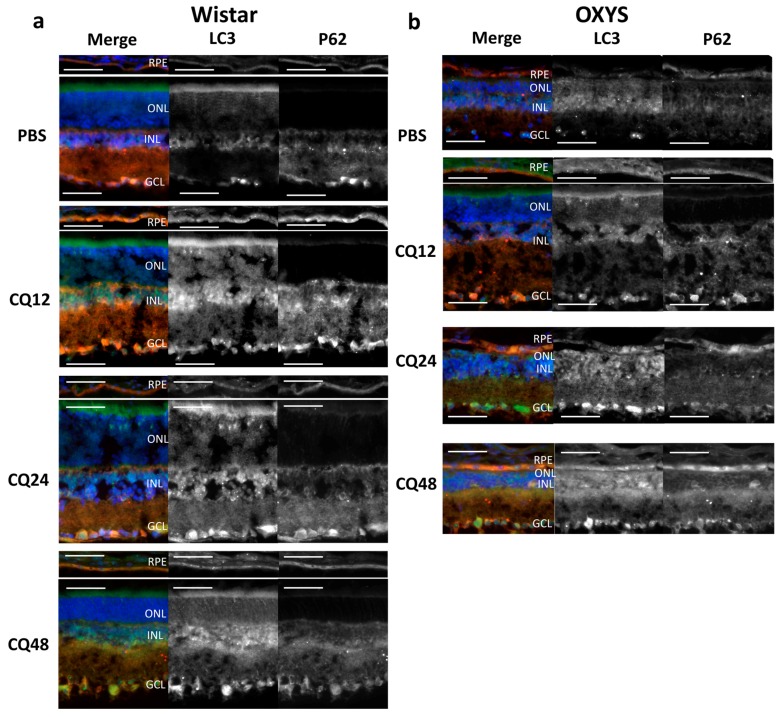
Representative images of retinal layers double-stained with antibodies against LC3 (green) and p62 (red) in Wistar (**a**) and OXYS (**b**) rats. Cell nuclei were stained with DAPI. Scale bar = 50 µm. RPE, retinal pigment epithelium; INL, inner nuclear layer; ONL, outer nuclear layer; GCL, ganglion cell layer; and IPL, inner plexiform layer.

**Figure 4:**
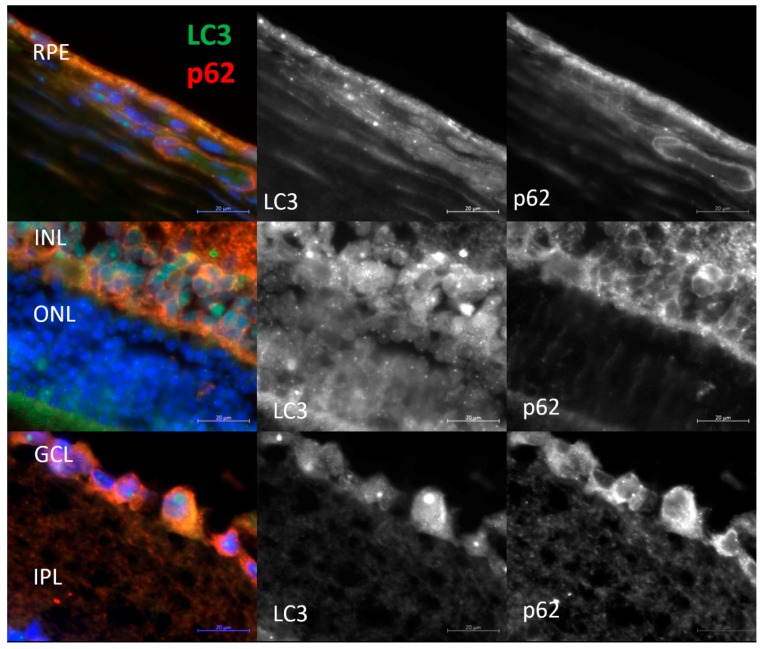
Representative enlarged images of retinal layers double-stained with antibodies against LC3 (green) and p62 (red). Cell nuclei were stained with DAPI. Scale bar = 20 µm. RPE, retinal pigment epithelium; INL, inner nuclear layer; ONL, outer nuclear layer; GCL, ganglion cell layer; and IPL, inner plexiform layer.

Methods of immunohistochemistry can detect the localization of a protein and evaluate its amount in individual layers of the retina by means of the intensity and area of the signal. One way to determine the level of autophagic activity is to evaluate the number, area, and intensity of LC3^+^ granules or cells in an autophagy-blocking or -inducing experiment. The number of autophagosomes in cells at baseline reflects a difference between the rate of their formation and the rate of fusion with lysosomes. Blocking the autophagosome–lysosome fusion allows us to evaluate the rate of formation of new autophagosomes. The ratio of the numbers of autophagosomes in groups with and without CQ reflects the ability of cells to remove newly forming autophagosomes [27] and serves as a measure of autophagic activity. Thus, the amount and/or intensity of LC3^+^ vesicles in groups of combined exposure to CQ with fasting for 12, 24, or 48 h in the corresponding fasting groups without exposure to CQ and their ratio will reflect the rate of autophagosome formation and the rate of removal of newly formed autophagosomes. It should be noted that enumeration of visible LC3^+^ vesicles (diameter >0.5 µm) did not reveal statistically significant differences between the groups with autophagy induction and those with autophagy blocking. Because the size of most autophagosomes is below the resolution limit of standard fluorescence microscopy (<0.5 µm), characteristics of the LC3^+^ signal intensity in different layers of the retina were used as a criterion for assessing the magnitude of autophagy induction. Representative images of immunostaining of LC3 and p62 in retinal layers are shown in Figure 3 and Figure 4 (enlarged images). 

**Figure 5:**
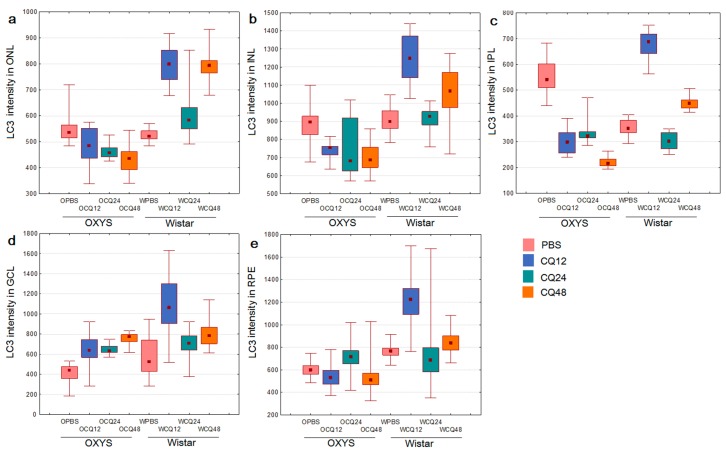
Changes in the amount of LC3^+^ autophagosomal vesicles in different layers of the retina upon modulation of autophagy in OXYS and Wistar rats: (**a**) in ONL; (**b**) in INL; (**c**) in IPL; (**d**) in GCL; (**e**) in RPE. Medians, the 25–75% interquartile range (bars), and Min–Max (error bars) are presented.

Evaluation of LC3B content in the retina of 16-month-old OXYS and Wistar rats by immunohistochemistry in the autophagy modulation experiment suggested that the LC3 signal intensity in the retina of Wistar rats increased after the exposure to CQ (Figure 5). Therefore, the median of the LC3 signal intensity in the RPE of Wistar rats by 12 h of fasting against the background of inhibition (WCQ12 group) increased and became higher in the outer nuclear layer (ONL) and RPE by 60%, in the inner nuclear layer (INL) by 40%, and in the inner plexiform layer (IPL) and ganglion cell layer (GCL) twice that in Wistar rats without inhibition (WPBS12). By contrast, in OXYS rats, the LC3 signal intensity did not change after CQ exposure or even slightly decreased in the ONL, INL, and IPL. In OXYS rats, LC3 signal intensity increased in the ganglion layer in all CQ groups and in the RPE layer in the CQ24 group, although this increase was less than that in Wistar rats. A reduced number of autophagosomes in cells after the blockage of autophagosome–lysosome fusion is characteristic of neurodegenerative diseases [27]. Moreover, the ratios of LC3^+^ signal intensities in groups with and without CQ were higher for Wistar rats in all the layers, indicating a decrease in the rate of removal of autophagosomes in OXYS rats.

**Figure 6:**
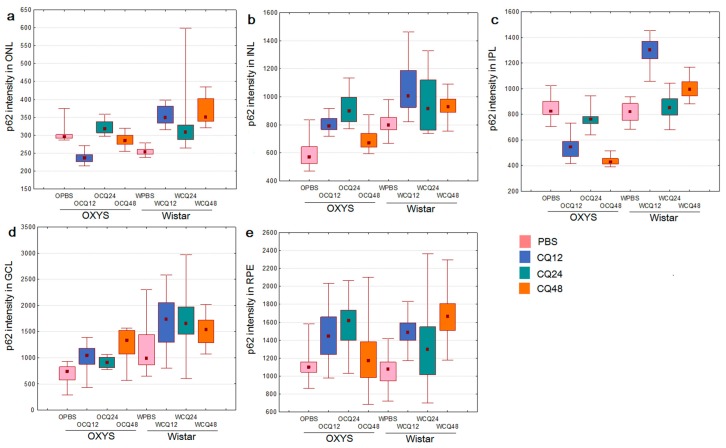
Changes in the p62 content of different layers of the retina upon modulation of autophagy in OXYS and Wistar rats: (**a**) in ONL; (**b**) in INL; (**c**) in IPL; (**d**) in GCL; (**e**) in RPE. Medians, the 25–75% interquartile range (bars), and Min–Max (error bars) are presented.

p62 is an autophagy-related receptor that links ubiquitinated cargo, including damaged organelles and potentially toxic protein aggregates, to LC3 and is delivered to autophagosomes. Therefore, increased autophagic flux generally leads to p62 downregulation, whereas p62 accumulates when autophagic degradation is inhibited [27]. We did not notice significant changes in the amount of the p62 protein during fasting. Nevertheless, during the inhibition of autophagy by CQ, some differences were identified between 16-month-old OXYS and Wistar rats. The immunoreactivity of p62 increased during CQ exposure in the RPE and INL of Wistar and OXYS rats (Figure 6). In contrast to Wistar rats, p62 signal intensity decreased in the CQ48 group of OXYS rats. The intensity of the p62 signal in the ONL after the exposure to CQ increased only in Wistar rats. Similar changes in the intensity of the LC3 and p62 fluorescent signals after exposure to CQ were observed in the GCL and IPL. In the GCL, upon exposure to CQ, the amount of LC3^+^ and p62^+^ granules increased in the rats of both strains; however, in OXYS rats, this effect was significantly weaker. In the IPL, the changes were multidirectional: in OXYS rats, LC3 and p62 contents decreased, whereas in Wistar rats, they increased. Thus, we found that with the progression of AMD signs in OXYS rats, both the rate of formation of autophagosomes and the rate of removal of newly formed autophagosomes decreased.

**Figure 7:**
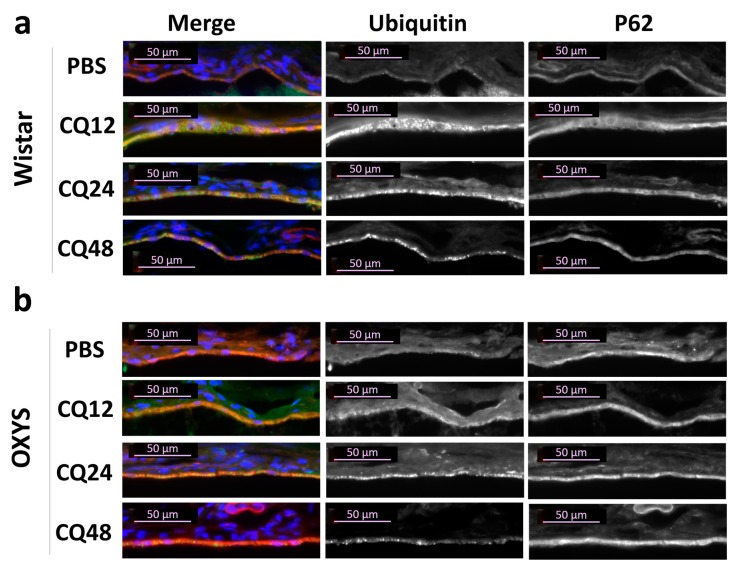
Representative images of RPE cryosections immunostained by ubiquitin (green) and the colocalization of ubiquitin with p62 (red) in (**a**) Wistar and (**b**) OXYS rats. Immunostaining for ubiquitin uncovered an increase of ubiquitin-positive granules in RPE cells of rats treated with CQ. Cell nuclei were stained with DAPI. The scale bar is 50 µm.

Ubiquitination is an important modification implicated in signaling transduction and protein degradation. As a cytosolic ubiquitin-binding protein, p62 regulates proteostasis by mediating the transient aggregation of ubiquitinated proteins under stress conditions. The presence of ubiquitin-positive particles and their colocalization with p62 were examined immunohistochemically in the RPE cells. Exposure to CQ led to the appearance of ubiquitin-positive granules in RPE (Figure 7), which were not detected in the RPE cells of intact and fasting rats (Figure S1). However, these granules at low level were found in PBS groups, suggesting that they could mark the aggresome-like induced structures (ALIS), provoked by stress condition. ALIS have been described as transient aggregations of ubiquitinated proteins in the cytosol, an early event in cellular adjustment to altered proteostasis under stress [28]. Compared to PBS, CQ significantly increased the number of ubiquitin-positive granules, as well as their size, in RPE of OXYS and Wistar rats (Figure 7 and Figure S1). Unlike ubiquitin, the immunofluorescence of p62 protein remained homogeneous in RPE cells after exposure to CQ despite a strong increase in immunoreactivity (Figure 7).

### 2.4. Impaired Autophagic Flux in RPE Cells of OXYS Rats Revealed by Transmission Electron Microscopy (TEM) 

**Figure 8:**
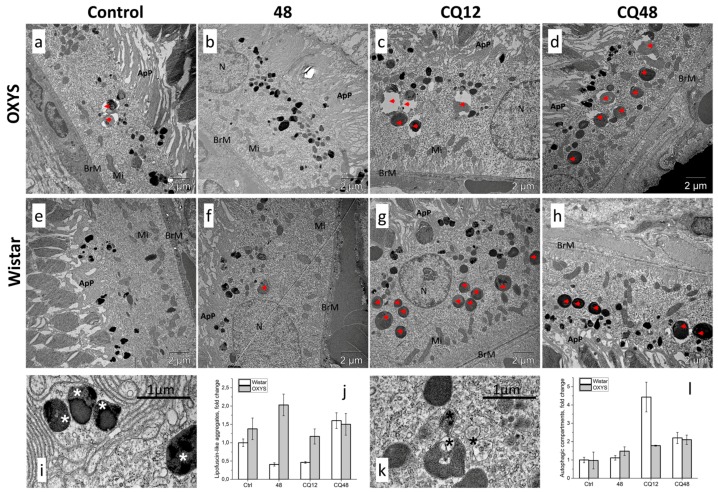
The effects of autophagy modulation on ultrastructure of RPE cells of 16-month-old (**a**–**d**) OXYS and (**e**–**h**) Wistar rats. Exposure to CQ significantly increased the number of autophagic compartments and phagosomes (red arrowheads) in the basal part of the RPE cell in (**g**) Wistar rats and to a lesser extent in (**c**) OXYS rats, indicating a decrease in the autophagic activity in OXYS rats. Lipofuscin-like aggregates (white asterisk in (**i**)) and autophagic compartments (black asterisk in (**k**)) were manually counted by (**j**,**l**) three different individuals. N, nucleus; Mi, examples of mitochondria; ApP, apical processes; BrM, Bruch’s membrane.

TEM is the only method that allows investigators to study the morphology of autophagosomal–lysosomal structures at nanometer resolution and to determine their location relative to other cellular structures. Using TEM, one can observe different stages of autophagy at the morphological level: from the formation of the phagophore to the stage of fusion of autophagosomes with lysosomes. Counting of such structures and identifying the predominant stage make it possible to draw conclusions about the presence or absence of autophagy disturbances. To assess the amount of lipofuscin-like aggregates and autophagic compartments and confirm the effective blockage of autophagy by CQ, we evaluated the ultrastructure of RPE cells in the retina of 16-month-old Wistar and OXYS rats from the following groups: control, 48 h fasting, CQ, and CQ48. In fasting rats, the amount of autophagosomal–lysosomal structures in RPE cells was slightly higher than that in the control rats, while the sizes of these structures did not change. The increased level of lipofuscin-like aggregates in OXYS rats were found (Figure 8j). A comparison of the ultrastructure of RPE cells of control rats and those receiving CQ indicated an increase in the amount and size of autophagosomal–lysosomal structures during the inhibition of autophagy (Figure 8c,d,g,h). The counting of autophagic compartments has revealed an increase in their number in groups treated with CQ. The increase was more pronounced in Wistar rats.

**Figure 9:**
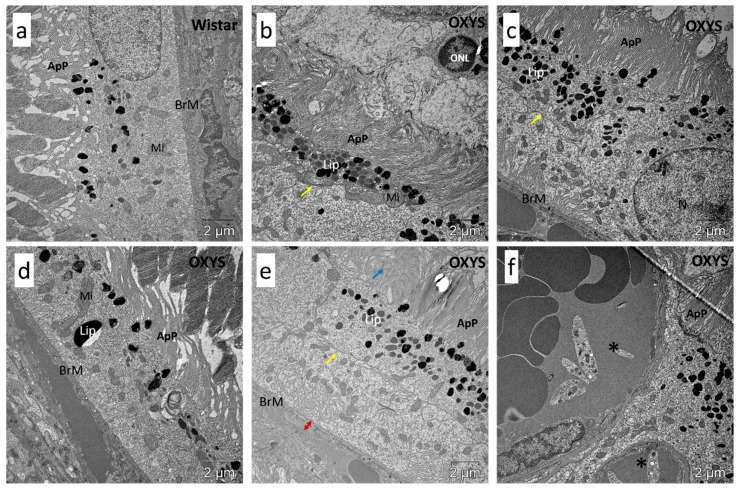
Ultrastructure of RPE cells of 16-month-old rats. Examples of degenerative changes in RPE of OXYS rats compared to (**a**) Wistar rats: (**b**) autophagic RPE cell death; (**c**) emergence of the second layer of RPE cells; (**d**) large lipofuscin granule accumulation; (**e**) degeneration of apical processes and thickening of Bruch’s membrane; (**f**) the growth of choriocapillaris through the RPE layer. N, nucleus; Mi, examples of mitochondria; ApP, apical processes; Lip, lipofuscin granules; BrM, Bruch’s membrane; ONL, outer nuclear layer; yellow arrow, the membrane between two layers of cells; blue arrow, degeneration of apical processes; red arrow, thickening of Bruch’s membrane; asterisks, choriocapillaris.

In Wistar rats, the CQ treatment significantly increased the number of large phagosomes (diameter ≥1 µm) containing the outer segments of the photoreceptors in the basal part of the RPE cell. By contrast, in OXYS rats, we noted a significantly lower number of autophagosomal–lysosomal structures after the CQ treatment, pointing to a reduced rate of autophagic flux and phagocytosis in the RPE of OXYS rats. Moreover, the lumens of these structures in OXYS rats were dilated, unlike in Wistar rats. In addition, in the OXYS rats of all groups, degenerative changes in individual sections of the pigment epithelium were observed: local instances of thickening of the Bruch basement membrane, fibrotic changes in the underlying layer of the choroid, accumulation of lipofuscin granules, emergence of a second layer of RPE cells, the growth of choriocapillaris, degeneration of apical processes, and signs of cell death of the autophagic type (Figure 9 and Figure S2).

## 3. Discussion

Here we describe a link between an impaired reactivity of the autophagy and AMD-like retinopathy development and consider its possible mechanisms, according to the results of transcriptome analysis. 

In a recent review by Wang and colleges [29], autophagy dysfunction in RPE cells, cellular senescence, and the abnormal immunoinflammatory response are discussed as the main drivers of AMD development. All three processes interact with one another both in a stimulating and inhibitory manner. For example, autophagy dysfunction accompanied by lipofuscin accumulation and increased reactive oxygen species can activate inflammatory reactions, by promoting low-intensity inflammation and accelerating RPE cell senescence [5]. Nonetheless, inhibition of autophagy mediated by Aβ decreases the levels of IL-1β, IL-6, IL-8, IL-12b, NLRP3, and TNF-α [30], suggesting that autophagy dysfunction may also inhibit inflammation. Another article [26] presents convincing arguments in favor of the leading role of lysosome-mediated pathways in the pathogenesis of irreversible blinding diseases. Both points of view find experimental confirmation in the OXYS rat model of AMD-like retinopathy. In the present study, we demonstrated that the development of AMD-like retinopathy in OXYS rats is accompanied by retinal transcriptome changes affecting genes involved in autophagy. These genes are associated with kinase activity, immune processes, and FoxO, mTOR, PI3K-AKT, MAPK, AMPK, and neurotrophin pathways at preclinical and manifestation stages, as well as vesicle transport and processes in lysosomes at the progression stage. Moreover, our bioinformatic gene network analysis of autophagy-related DEGs showed their association at the age of 3 months with an immunoinflammatory response and at the age of 18 months with processes in lysosomes. Besides, at all ages, the expression levels of genes related to the GO term “fusion regulation” were found to be altered. Previously, analysis of RNA-Seq data has revealed significant downregulation of immune-system genes in OXYS rat retinas [22].

There is no doubt that gradual degeneration of the RPE is a precursor to pathological changes in the outer retina. The phagocytic function of the RPE and the visual cycle are linked via the noncanonical form of autophagy. The RPE internalizes and digests up to 10% of POSs (by volume) daily. If autophagic dysfunction takes place in RPE cells, then POSs cannot be fully degraded, leading to lipofuscin deposition and drusen formation. Several POS components contribute to the buildup of lipofuscin-like material in the RPE cell: aggregates of bis-retinoid A2E as well as chemically modified compounds such as malondialdehyde and 4-hydroxynonenal [26]. Their impaired clearance and processing by the autophagy pathway increase proteolytic and oxidative stress, thereby causing irreversible damage to RPE cells as well as the development of AMD. As demonstrated by Mitter and colleges [6] the dysregulated autophagy in the RPE is associated with increased susceptibility to oxidative stress, whereas increased autophagic flux protects the RPE from oxidative damage. The deletion of *Rb1cc1* (also known as *Fip200*) acting as an inducer of autophagy results in multiple autophagy defects, age-related degeneration of RPE cells, sub-RPE deposition of inflammatory and oxidatively damaged proteins with migration of activated microglial cells, and occasional foci of choroidal neovascularization [7]. Age-related retinal degeneration has also been observed in mice with RPE-specific deletion of *Atg5* or *Atg7*. Early AMD-like RPE defects in these mice include uneven RPE thickness, RPE hypertrophy or hypotrophy, pigmentary irregularities, choroidal neovascularization, and necrosis [31]. RPE-specific deletion of *Atg5* in mice disrupts POS degradation, diminishes chromophore levels, and worsens visual function [12]. In the present study, we detected autophagosomal–lysosomal vacuoles with a large lumen in RPE cells of OXYS rats exposed to CQ. This phenomenon may be a consequence of a chronic imbalance of ion homeostasis, possibly causing water influx and swelling of lysosomes [8]. Recently, Lei and colleges [32] reported that the inhibition of the autophagy-related proteins resulted in an increase in lipofuscin-like autofluorescence of ARPE-19 cells when cells are fed with POSs, confirming the role of autophagy in the fate of RPE lipofuscin degradation. Manipulation of autophagy in RPE cells is being explored as a potential way to regulate early lipofuscin formation [32]. Moreover, a neuroprotective effect of fasting on retinal ganglion cell survival has been documented [33], suggesting that short-term food restriction is a promising intervention against retinal neurodegenerative disorders. 

Autophagy represents a cytoprotective self-adaptation mechanism allowing cells to cope with stressful conditions. An ischemic insult in the retina triggers a biphasic and reperfusion time-dependent response of the autophagy process [33]. Increased neuronal loss is observed in mice with a genetic impairment of basal autophagy owing to heterozygous ablation of a positive modulator of autophagy, Ambra1 (Ambra1^−/+^) [33]. Food deprivation induces autophagic flux in the retina [33]. We found that 24 h of food deprivation increased the expression of *Atg5*, *Atg7*, *Becn1*, *Gabarapl1*, *Nbr1*, and *p62* at the transcriptional level in the retina of OXYS and Wistar rats. A shorter fasting period (12 h) did not alter the expression of these genes. These results are consistent with findings of study [34], which noted activation of autophagy in all the retinal layers of mice subjected to food restriction for 24 h. In our study, the upregulation of autophagy-related genes disappeared by 48 h in the retina of OXYS rats but remained high in Wistar rats, meaning decreased reactivity of the autophagy system in OXYS rats. The changes in transcriptional activity after autophagy inhibition by CQ were demonstrated for the first time. We propose that this effect is mediated by the impact of CQ on the signal complex anchored on lysosomal v-ATPase, which reversibly binds to mTORC1 and transcription factor TFEB. Possibly, CQ affects mTOR-dependent phosphorylation of TFEB, the level of which determines the degree of translocation of TFEB into the nucleus to activate the expression of genes involved in the formation of lysosomes and the initiation of autophagy [35]. Thus, the dynamics and reactivity of autophagy in senescence-accelerated OXYS rats differ from those of Wistar rats: at the transcriptional level, the response to modulation of autophagy is less pronounced in OXYS rats than in Wistar rats.

The ubiquitin–proteasome system (UPS) and the lysosomal/autophagosomal degradation system maintain cellular proteostasis by recognizing the cytoplasmic material intended for degradation and recycling it. Ubiquitinated proteins are directed to the proteasomal degradation. However, the capacity of protein degradation by UPS is limited. Unlike the UPS, autophagy can remove large cell aggregates and obsolete organelles. Multifunctional protein P62 connects the UPS and autophagy clearance systems: through ubiquitin-binding domain P62 recognizes ubiquitinated protein aggregates and then using LC3-binding domain P62 shuttles with tagged material to autophagosome [36]. Double knockout NRF-2/PGC-1α mice develop dry AMD-like phenotype on the background of significant age-dependent RPE degeneration, accumulation of the oxidative stress markers, increased levels of protein aggregation markers (ubiquitin and p62) and the increased size of autolysosomes in RPE cells, indicating an insufficient rate in autophagic and proteasomal clearance [36]. In our study, we showed that RPE cells respond to autophagy inhibition by CQ with a significant increase in the content of ubiquitin-positive granules that are not found in intact groups. We believe that CQ enhanced ALIS formation.

Many studies suggest that the decline of autophagy with age contributes to a number of age-related diseases [27]. Nevertheless, there is currently no consensus on whether autophagic activity increases or decreases with age and disease. Apparently, this phenomenon depends on such factors as cell type, disease, or a specific stage of the disease [26]. We have reported that the basal level of autophagy is elevated at the early stage of retinopathy and declines at progressive stages [16]. The autophagy process strengthens in two mouse models of AMD and in early-onset human AMD samples but declines in late AMD [6]. In the present study, we demonstrated that retinal autophagy flux is lower in OXYS rats, thereby reducing the capacity of retinal cells to cope with the elevated proteolytic stress in progressive stages of disease. As one of the reasons for the decreased reactivity of autophagy in the retina of OXYS rats, we can propose accumulation of senescent RPE cells. These cells lead to cellular dysfunction and promote the senescence of neighboring cells by secreting the so-called senescence-associated secretory phenotype [29]. The converse is also possible: autophagy deregulation contributes to the accumulation of senescent cells. Previously, we have observed altered RPE cell morphology in OXYS rats already at age 3 months: enlargement, flattening, a loss of the hexagonal shape, accumulation of lipofuscin granules, and multinucleation, which are employed as morphological markers of senescent cells [15]. Early exhaustion of autophagy processes is also detectable in the kidneys of OXYS rats [37], consistently with their accelerated-senescence phenotype. 

Taken together, this work and our related studies [16,17,21] prove a malfunction of autophagy in the retina of OXYS rats.

## 4. Materials and Methods 

### 4.1. Ethics Statement

All animal procedures were in compliance with the Association for Research in Vision and Ophthalmology statement for the Use of Animals in Ophthalmic and Vision Research and the European Communities Council Directive 86/609/EES. All manipulations of the animals were approved by Scientific Council 9 of the Institute of Cytology and Genetics, SB RAS, according to The Guidelines for Manipulations with Experimental Animals (the decree of the Presidium of the Russian Academy of Sciences No. 12000-496 of April 2, 1980).

### 4.2. Animals and Treatments

Male Wistar and senescence-accelerated OXYS rats were purchased from the Center for Genetic Resources of Laboratory Animals at the Institute of Cytology and Genetics, SB RAS (RFMEFI61914X0005 and RFMEFI61914X0010). The OXYS strain was derived from the Wistar strain of rats at the Institute of Cytology and Genetics as described earlier [38]. The rats were kept under conventional conditions on the 12:12 h light/dark cycle (08:00 lights on) and had ad libitum access to food pellets (PK-120-1; Laboratorsnab, Ltd., Moscow, Russia) and water unless stated otherwise. All experiments were approved by (and conducted in accordance with the guidelines of) the Ethics Committee on animal testing of the Institute of Cytology and Genetics, Novosibirsk, Russia.

OXYS and Wistar rats at the age of 4 and 16 months were randomly distributed into treatment and control groups (*n* = 6 of each genotype per group). The rats consumed feed ad libitum, fasted for 12, 24, or 48 h, or fasted during four daily intraperitoneal injections of CQ (50 mg/kg, Sigma-Aldrich, St. Louis, MO, USA) with the last injection administered 3 h before euthanasia. Phosphate-buffered saline (PBS)-treated rats served as a control for the CQ treatment. To avoid circadian issues, all rats were euthanized at 11:00–13:00 h sequentially, where rats from different groups followed each other. The rats were euthanized by CO_2_ asphyxiation and decapitated. 

The retina from the left eye of each rat (*n* = 6 per group) was separated from the other tissues, placed in a microcentrifuge tube for mRNA isolation, and frozen in liquid nitrogen and stored at −70 °C before the analysis. 

### 4.3. Functional Analysis of RNA-Seq Data

RNA-Seq data from the retinas of OXYS and Wistar rats at 20 days and 3 and 18 months of age (*n* = 3 animals per genotype and age) were obtained and processed as described elsewhere [22,23]. Because we were interested only in the DEGs associated with autophagy, their networks, and the interdependent signaling pathways involved, we increased the differential expression threshold to *p* < 0.001, guided by the considerations outlined in another study [39]. The list of DEGs associated with autophagy was compiled according to the AUTOPHAGY database (http://www.tanpaku.org/autophagy/), GO (http://geneontology.org/), DAVID (https://david-d.ncifcrf.gov/tools.jsp), and KEGG. To identify the GO terms and pathways associated with autophagy over-represented in a DEG list, the detected DEGs were subjected to functional enrichment analyses by means of the DAVID tool with a Benjamini *p*-value cutoff at <0.05. The gene interaction networks related to autophagy were identified on the GeneMANIA web server (http://www.genemania.org/) with default parameters.

### 4.4. Quantitative Reverse-Transcription PCR (qPCR)

The relative amount of an mRNA of interest was measured by qPCR. Sequences of primers were designed in Primer 3 software (National Center for Biotechnology Information, USA); exon-spanning primer sets including large introns were used to eliminate the detection of residual genomic DNA (Table 2). 

Housekeeping gene *Rpl30* served as a reference gene. Each pair of primers was tested by qPCR with the fluorescent SYBR Green dye on thermocycler CFX96 (Bio-Rad, Hercules, USA) followed by determination of the melting temperature of the reaction product. Oligonucleotides were synthesized by Biosan (Biosan, Novosibirsk, Russia). Extraction of total RNA from freshly frozen tissue was performed with the Tri-reagent (Sigma-Aldrich). Reverse transcription was performed by means of M-MLV Reverse Transcriptase (Promega, Madison, WI, USA).

In each experiment, cDNA samples with specific primers (four technical replicates per each sample) and *Rpl30* gene (also four technical replicates per sample) were placed on the same plate; to construct a calibration curve, a standard cDNA template (obtained via mixing of all cDNA samples) and serial fourfold dilutions (1:1, 1:4, 1:16, and 1:64) with the same primers (two replicates per dilution) were placed on each plate. A relative mRNA level of a gene was determined via calibration curves obtained based on dilutions of the standard cDNA. The result for each gene of interest was normalized to the *Rpl30* gene data.

### 4.5. Immunohistochemistry

The right eyes of rats (*n* = 5 per group) were excised and fixed in fresh 4% paraformaldehyde in PBS for 2 h, washed three times in PBS, and then cryoprotected in graded sucrose solutions (10%, 20%, and 30%). Posterior eyecups were embedded in Killik (Bio-Optica, Milano, Italy), frozen, and stored at −70 °C. Tissue slices (10 µm thick) were prepared on a Microm HM-505N cryostat at −20 °C, transferred onto Polysine® glass slides (Menzel-Glaser, Waltham, USA), and stored at −20 °C. After several washes in PBS with 0.1% of Triton X-100 (PBST), the slices were incubated for 1 h in 5% BSA in PBST, followed by overnight incubation at 4 °C with the rabbit antibodies to LC3A/B, ubiquitin (1:150 dilution in PBST with 1% of BSA, Abcam) and the mouse antibodies to p62 (1:150 dilution, Abcam). After three washes in PBST, the tissue slices were incubated with the secondary antibodies conjugated with Alexa Fluor® 488 or Alexa Fluor® 564 (Abcam, Cambridge, UK) at a dilution of 1:300 for 1 h and next washed with PBST. The slices were coverslipped with the Fluoroshield mounting medium supplemented with 4′,6-diamidino-2-phenylindole (DAPI, Abcam) and examined under an Axioplan 2 microscope (Carl Zeiss SMT GmbH, Oberkochen, Germany). For acquisition of all images, the imaging parameters were the same. Three tissue slices (technical replicates) were analyzed per animal. Using Zen LE software (Carl Zeiss SMT GmbH, Oberkochen, Germany), regions of interest were set up to define individual retinal layers, and data on the average fluorescence intensity of Alexa Fluor were collected, reflecting the amount of protein.

### 4.6. TEM

For electron microscopic examination, small samples from the retina of the right eye (*n* = 1 per group) were fixed with 2.5% glutaraldehyde in 0.1 M cacodylate buffer for 1 h at room temperature, washed twice in the buffer and water (MilliQ, Merck Millipore, Burlington, USA), then postfixed with 1% aqueous solution of osmium tetroxide with a few crystals of potassium ferricyanide (K_3_(Fe(CN)_6_)) for 1 h at room temperature and finally incubated in 1% aqueous solution of uranyl acetate overnight. Next day, the samples were dehydrated in ethanol series and acetone and embedded in the Agar 100 Resin (Agar Scientific) blocks. Resin blocks were polymerized at 60 °C for 3 days. Ultrathin (70 nm) sections were prepared with Leica Ultracut ultra-microtome (Leica Microsystems GmbH, Wetzlar, Germany). The tissue slices were examined under a JEOL JEM-100SX transmission electron microscope (JEOL Ltd., Tokyo, Japan) at 60 kV at the Microscopy Center of the Institute of Cytology and Genetics SB RAS. Lipofuscin-like aggregates and autophagic compartments were manually counted by three different individuals as described in [36].

### 4.7. Statistical Analysis

The data were subjected to ANOVA (Statistica 8.0 software, Statsoft, Tulsa, OK, USA). ANOVA followed by the post hoc Newman–Keuls test was performed when ANOVA assumptions were met; otherwise (i.e., in the absence of a normal distribution of the data in the groups under study), nonparametric Kruskal–Wallis ANOVA was carried out followed by multiple comparisons of mean ranks for all groups. The independent variables for two-way ANOVA were genotype and treatment. The data were presented as mean ± SD. The differences were considered statistically significant at *p* < 0.05.

## 5. Conclusions

In this study, we showed that dysregulation of the autophagy transcriptome network in retinal cells takes place already at early stages of AMD-like retinopathy. Using OXYS rats as a model of AMD, we demonstrated that aberrant capacity for upregulation of autophagic flux in response to metabolic stress in the retina accompanies the development of AMD-like retinopathy and may reflect an age-related decrease in the adaptability of retinal cells. Consequently, maintaining sufficient reactivity of autophagy in the retina may be considered a promising strategy to slow down AMD. 

## Figures and Tables

**Table 1:** Enriched gene ontology (GO) terms and Kyoto Encyclopedia of Genes and Genomes (KEGG) pathways (according to Database for Annotation, Visualization and Integrated Discovery (DAVID)) in the set of DEGs associated with autophagy in the retina of OXYS rats at ages 20 days and 3 and 18 months (Benjamini *p*-value < 0.05).

Category	20 Days	3 Months	18 Months
Biological processes	Autophagy, macroautophagy, regulation of autophagy, intracellular signal transduction, protein phosphorylation, response to oxygen-containing compound, response to organic substance, cellular response to stress, cell death, regulation of apoptotic process		Protein phosphorylation, response to organic substance
	TOR signaling, apoptotic process, autophagosome assembly, vesicle-mediated transport, neurogenesis	Response to lipid, peptidyl-amino acid modification, innate immune response, response to lipopolysaccharide, response to oxidative stress	Regulation of organelle organization, neurogenesis, response to nitrogen compound, response to light stimulus, Golgi to plasma membrane transport, histone H3 deacetylation
Molecular functions	Protein binding, protein serine/threonine kinase activity, ATP binding, protein kinase binding		
	Protein deacetylase activity	Transcription factor binding	Calcium-dependent cysteine-type endopeptidase activity
Cell compartments	Vacuolar membrane, cytoplasmic, membrane-bounded vesicle, vesicle membrane, Golgi apparatus		
	Autophagosome, endosome membrane, protein complex, TORC2 complex	Autophagosome, membrane coat, TOR complex, lysosome, endosome, AP-type membrane coat adaptor complex	Extracellular exosome
KEGG pathway	FoxO signaling, neurotrophin signaling, Toll-like receptor signaling, TNF signaling		
	mTOR signaling, PI3K-AKT signaling, MAPK signaling, AMPK signaling, regulation of autophagy	Lysosome, B cell receptor signaling, T cell receptor signaling, chemokine signaling	Endocytosis

**Table 2:** Primer sequences.

Title 1	Forward	Reverse
*Atg5*	ACCTCGGTTTGGCTTGGTTG	AGTATGGCTCTGCTTCTCGTT
*Atg7*	AGCCTGTTCATCCAAAGTTCT	CTGTGGTTGCTCAGACGGT
*Becn1*	GCGTCGGGGCCTAAAGAATG	CTCCTGGCTCTCTCCTGGTT
*Gabarapl1*	CCTCCGACCTCACTGTTGG	TGCCTCATTTCCCGTAGACAC
*Map1lc3b*	GGAGCTTCGAACAAAGAGTGG	TGCAGGCGCCTTCTAATTATCT
*p62*	CTGAGTCGGCTTCTGCTCCAT	ATCTTCTGTGCCTGTGCTGGA
*Nbr1*	TAGTCCCAGAAGTGGCAGGA	ATTGTGGTGCCTTGAGTGGT
*Rpl30*	ATGGTGGCTGCAAAGAAGAC	CAAAGCTGGACAGTTGTTGG

## Supplementary Materials

Supplementary materials can be found at https://www.mdpi.com/1422-0067/20/19/4804/s1.

## Abbreviations

AMD	Age-related macular degeneration
ALIS	Aggresome-like induced structures
BSA	Bovine serum albumin
CQ	Chloroquine
DAVID	Database for Annotation, Visualization and Integrated Discovery
DEGs	Differentially expressed genes
GCL	Ganglion cell layer
GO	Gene Ontology
INL	Inner nuclear layer
IPL	Inner plexiform layer
KEGG	Kyoto Encyclopedia of Genes and Genomes
ONL	Outer nuclear layer
PBS	Phosphate-buffered saline
POS	Photoreceptors outer segments
RNA-Seq	High-throughput transcriptome sequencing
RPE	Retinal pigment epithelium
UPS	Ubiquitin-proteasome system
