# Supplementary material for: Disruptions of Autophagy in the Rat Retina with Age During the Development of Age-Related-Macular-Degeneration-like Retinopathy
Source: Int J Mol Sci. 2019 Sep 27;20(19):4804. doi: 10.3390/ijms20194804 (PMC6801580; doi:10.3390/ijms20194804)

Table S1. Autophagy-related differentially expressed genes (DEGs)

**Age**	**Autophagy-related DEGs**
20 days	Upregulated: *Akt3, Ap1b1, Ap3b2, Arhgap26, Atf4, Atg16l1, Atg16l2, Camk1g, Camkk1, Cbr1, Ddit4, Dnm1, Ehmt2, Elfn2, Flnb, Fos, Gabarapl1, Gapdhs, Grtp1, Hap1, Harbi1, Hdac4, Hif1a, Klhl6, Mapk12, Mapk13, Mapk14, Mapt, Nlrc3, Optn, Plcd3, Prr5l, Qsox1, Ralgapa2, Rnf5, Sh3bgrl2, Shroom3, Slc1a3, Stab2, Stk11, Stx17, Tbc1d5, Trim65, Tsga10ip, Ugt2b17, Ulk3*<br>Downregulated: *Actr2, Actr3, Alk, Alppl2, Bcl2, Cdk2, Cdkl5, Cdkn1b, Csnk1g3, Deptor, Dram2, Eif4e, Exoc2, Exoc5, Fam134b, Fgfr2, Gopc, Hdac9, Hunk, Ikbke, Itpr1, Klhl4, Lamp2, Lamp3, Mitf, Mtmr2, Ngf, Pik3cg, Pkn2, Plcb4, Ppp2r2b, Ppp2r3c, Ppp6c, Prkaa2, Prr5, Pten, Rab10, Rb1cc1, RGD1566251, Rps6ka3, Rps6ka6, Scfd1, Sesn3, Sirt1, Slc7a11, Snx2, Sos2, Tm9sf2, Tm9sf3, Tns1, Ube2d2, Ube2d3, Vamp7, Vps13a, Yes1*
3 months	Upregulated: *Ap2a2, Ap3b2, Atg7, Camk1g, Cdk17, Dnm1, Ehmt2, Fos, Gabarapl1, Hdac5, Hdac9, Hspa8, Kdr, Klhl18, LOC503238, Mapk12, Mlst8, Mtdh, Nlrc3, Npc1, Phf23, Pik3r2, Pip5k1c, Plcd3, S100a9, Sgk1, Sik1, Smpd1, Supt5h, Tbc1d5, Trappc9, Ugt2b17, Vps18, Vps39*<br>Downregulated: *Actr2, Alk, Ap1s3, Arhgef4, Capn13, Capns2, Cdk1, Cdkn1b, Eif2ak2, Foxo3, Gfap, Iars, Irgm, Krt76, Lamp3, LOC102554129, Lyn, Mx1, Mx2, Myd88, Nfkb1, Nfkbia, Nfkbiz, Pdk1, Rps6ka2, Sesn2, Sesn3, Sh3gl3, Shc3, Slc1a1, Slc1a4, Zc3h12a*
18 months	Upregulated: *Camk1g, Cdkl1, Hdac5, Hdac9, Mx2, Npas1, Ntrk2, Slc1a3, Stxbp1, Tbc1d5, Ugt2b17, Vps4b*<br>Downregulated: *Anxa7, Ap1s3, Arhgef4, Capn1, Capn2, Capn3, Capn5, Capns2, Cdk1, Dnm2, Ehmt2, Fgfr3, Lamp3, Lpin2, Mink1, Mtmr2, Pld2, Plk2, Ppp2r5a, Rab10, Ryr1, Smpdl3a, Ube2j2*
From 3 to 18 months	Upregulated with age in OXYS: *Alk, Arhgef4, Bcl2l1, Ephb2, Exoc3l4, Gfap, Hap1, Hspa2, Itpr3, Map2k1, Mapt, Npas1, Ntrk2, Prkcb, Prkcg, Prkcz, Ralgapa2, Rps6ka2, Sesn3, Shc3, Slc1a3, Tcirg1, Tnik, Tns3, Trim8*<br>Downregulated with age in OXYS: *Arhgap10, Atf4, Cdk16, Epha3, Fos, Hdac2, Ikbkb, Klhl18, Man2b1, Npas2, Plcd3, Scfd1, Sh3gl3, Sh3glb1, Sirt1, Tecpr1, TP53INP2, Vps39, Wac, Wdr45*<br>*Upregulated with age in Wistar: Actg1, Ap3b2, Atp6v0a1, Ehmt2, Hap1, Lpin2, Map3k6, Mapk13, Mapk14, Mapt, Mertk, Mink1, Mlst8, Pak1, Phf23, Prkcg, Ralgapa2, Ripk4, Tns3,*<br>*Downregulated with age in Wistar: Atf4, Dram2, Eif2ak2, Foxo3, Hdac9, Iars, Kdm4a, Mx1, Mx2, S100a9, Sesn2, Sh3gl3, Slc1a4, Wdr3*

Figure S2. Immunostaining for ubiquitin (green) uncovered an increase of ubiquitin-positive granules in RPE cells of rats treated with CQ. Cell nuclei were stained with DAPI. The scale bar is 20 µm.

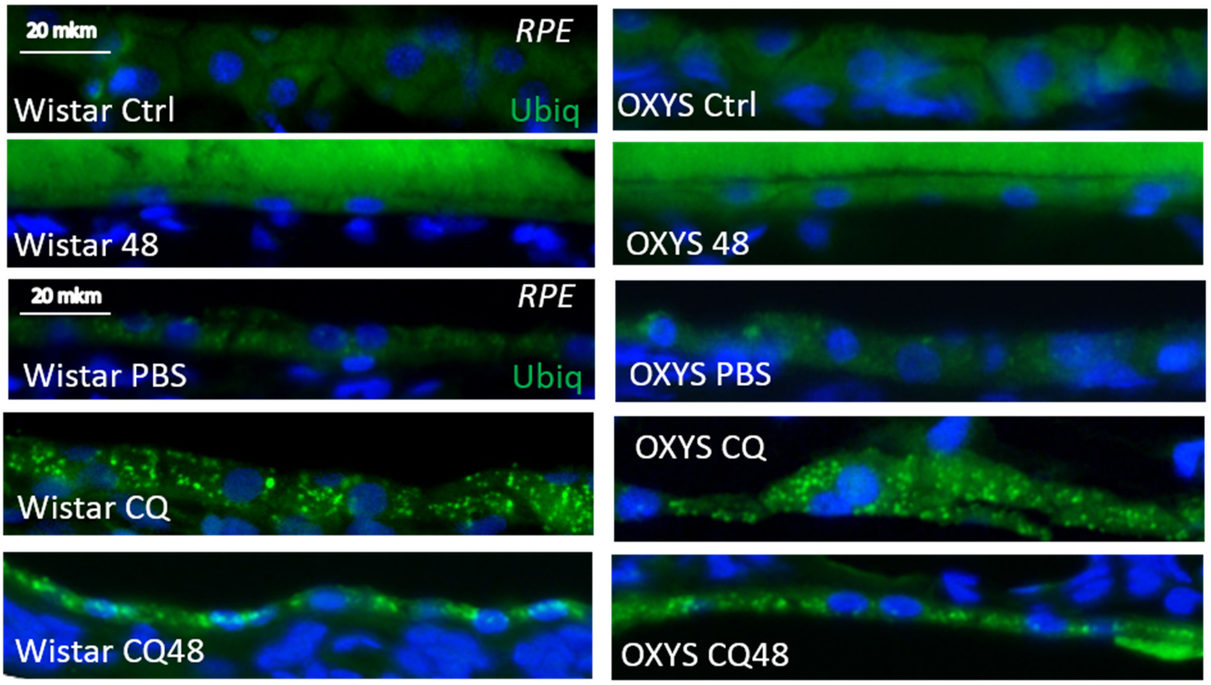

Figure S2. Representative TEM images assembly of ultrastructure of RPE cells in 16-month-old OXYS rat.

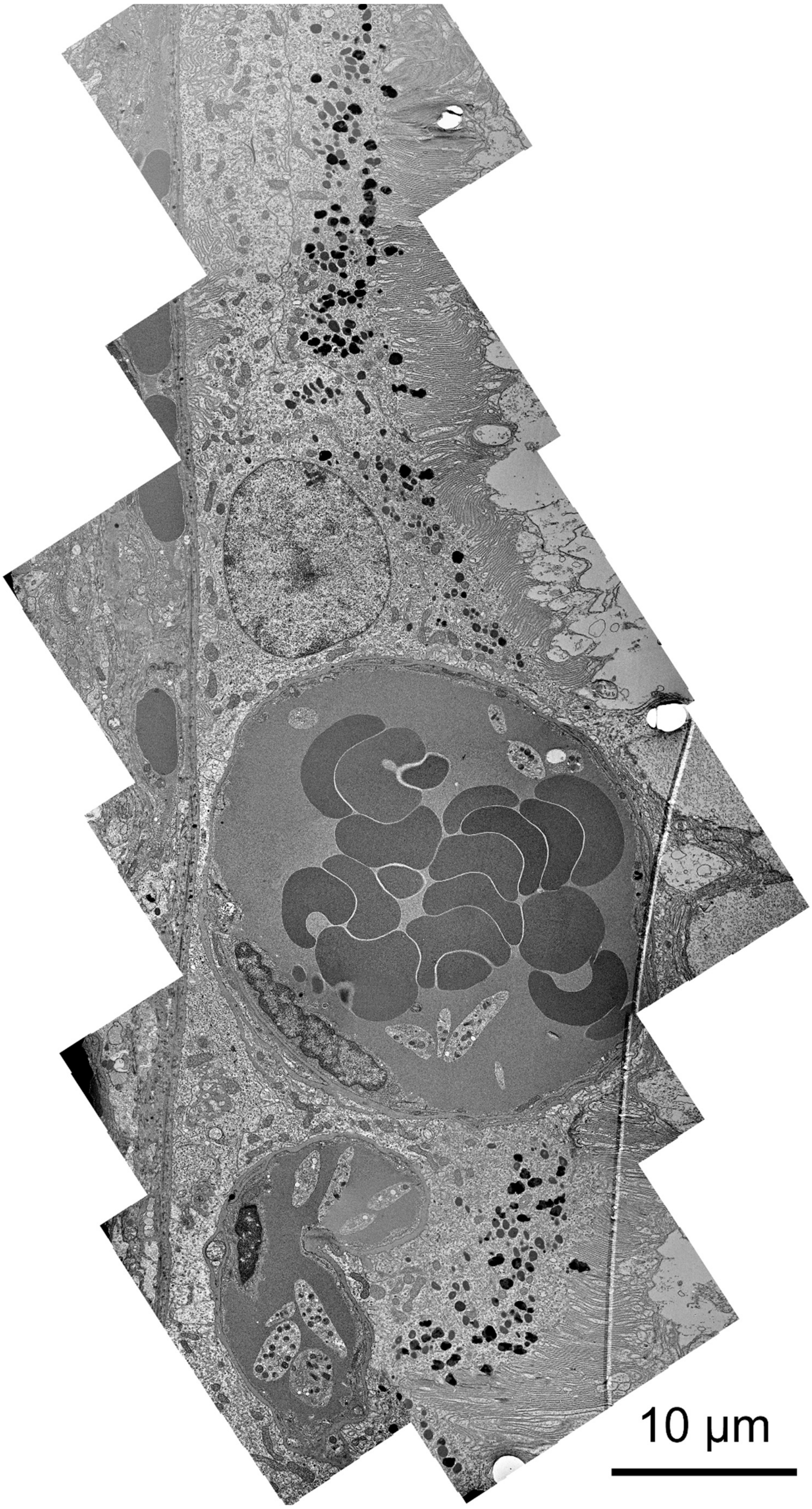

Supplement: Supplementary file 1 [file ijms-20-04804-s001.pdf]
